# Hepatic steatosis depresses alpha-1-antitrypsin levels in human and rat acute pancreatitis

**DOI:** 10.1038/srep17833

**Published:** 2015-12-04

**Authors:** Qian Wang, Jianjun Du, Pengfei Yu, Bin Bai, Zhanwei Zhao, Shiqi Wang, Junjie Zhu, Quanxin Feng, Yun Gao, Qingchuan Zhao, Chaoxu Liu

**Affiliations:** 1 Department of Surgery, Xijing Hospital of Digestive Diseases, Fourth Military Medical University, Xi’an, 710032, China; 2 Department of General Surgery, Huashan Hospital, Fudan University, Shanghai, 200040, China; 3 Department of Surgery, China PLA 323323 Hospital, Beijing, 100853, China; 4 Department of General Surgery, Huashan Hospital North, Fudan University, Shanghai, 201907, China; 5 Department of Surgery, Xijing Hospital of Digestive Diseases, Fourth Military Medical University, Xian, 710032, China

## Abstract

Hepatic steatosis (HS) can exacerbate acute pancreatitis (AP). This study aimed to investigate the relation between α1-antitrypsin (AAT) and acute pancreatitis when patients have HS. Using proteomic profiling, we identified 18 differently expressed proteins pots in the serum of rats with or without HS after surgical establishment of AP. AAT was found to be one of the significantly down-regulated proteins. AAT levels were significantly lower in hepatic steatosis acute pancreatitis (HSAP) than in non-HSAP (NHSAP) (P < 0.001). To explore the clinical significance of these observations, we measured the levels of AAT in the serum of 240 patients with HSAP, NHSAP, fatty liver disease (FLD), or no disease. Compared with healthy controls, serum AAT levels in patients with NHSAP were significantly higher (P < 0.01), while in patients with HSAP serum AAT levels were significantly lower (P < 0.01). Further studies showed that acute physiology and chronic health evaluation (APACHE-II) scores were negatively correlated with serum AAT levels (r = −0.85, P < 0.01). In conclusion, low serum levels of AAT in patients with HSAP are correlated with disease severity and AAT may represent a potential target for therapies aiming to improve pancreatitis.

Acute pancreatitis (AP) is an inflammatory condition of the pancreas^1^. AP will spontaneously resolve in most cases, but it can develop into severe AP (SAP), which can be fatal^2^. The overall mortality rate of SAP is approximately 20%. Several risk factors for SAP have been reported including alcohol, obesity^3^, opioid drugs^4^, and non-alcoholic fatty liver disease (NAFLD), also termed hepatic steatosis (HS)^5^,^6^. Rather than representing a specific disease, NAFLD is a clinical phenomenon characterized by inflammation, necrosis, and degeneration of liver cells^7^. NAFLD affects 10 to 24% of the general population in many countries^8^,^9^,^10^.

The mechanisms by which fatty liver disease (FLD) aggravates pancreatitis remain to be elucidated; however, several potential processes have been proposed. FLD is commonly accompanied by hyperlipidemia^11^, which has been reported to cause microcirculatory disturbances, oxidative stress, free radical accumulation, and/or acinar necrosis^12^,^13^,^14^,^15^. Hyperlipidemia may also reduce red blood cell velocity, and thus increase hemoglobin-oxygen affinity in the microcirculation, which can induce tissue hypoxia^12^. In addition, increasing free radical accumulation and oxidative stress may promote AP^13^,^14^,^15^,^16^, and interstitial release of triglyceride degradation products may exacerbate cellular disruption^15^. FLD may indeed exacerbate pancreatitis through a variety of mechanisms.

Alpha-1antitrypsin (AAT) is a tissue-diffusible and water-soluble glycoprotein. Over 80% of AAT is synthesized and secreted by hepatocytes, macrophages, and some cancer cells^17^. As serum levels of AAT increase in response to tissue injury, this glycoprotein is considered an acute-phasereactant^18^,^19^. In addition to inhibiting neutrophil elastase, chymase, and trypsin^19^, AAT also inhibits proteolytic enzymes such as cathepsin-G and proteinase-3 (PR3)^20^. However, under inflammatory conditions, AAT activity has been reported to be reduced. For example, hydrogen peroxide, a component of cigarette smoke, can inactivate AAT^21^. Severe AAT deficiency leads to an imbalance between proteinases and inhibitors, promoting the development of diseases such as (chronic obstructive pulmonary diseases) COPD, cirrhosis, and hepatocellular carcinoma^22^.

Imbalance in the synthesis and degradation of proteolytic enzymes and antiproteases has also been implicated in the development of AP. Elevated serum levels of AAT have been detected in AP^23^, and AAT may represent a diagnostic marker of the early phase of AP^24^,^25^,^26^. However, the influence of HS on AAT levels in the acute phase of pancreatitis is not known.

In order to further investigate the relationship between HS and pancreatitis, we established rat models of HSAP and NHSAP. We used proteomic analysis to evaluate the changes at the protein level in the serum of animals with HS during establishment of AP and found AAT to be significantly depressed in HSAP model animals. To further confirm the clinical significance of these findings, we measured the AAT levels in the serum of patients and found serum AAT levels to be significantly lower in HSAP patients than in NHSAP patients. The depressed serum levels of AAT in HSAP patients were also correlated with disease severity, potentially highlighting a mechanistic role for AAT in the exacerbation of pancreatitis caused by HS.

## Results

### Establishment and characterization of the rat models of HSAP and NHSAP

**Figure 1:**
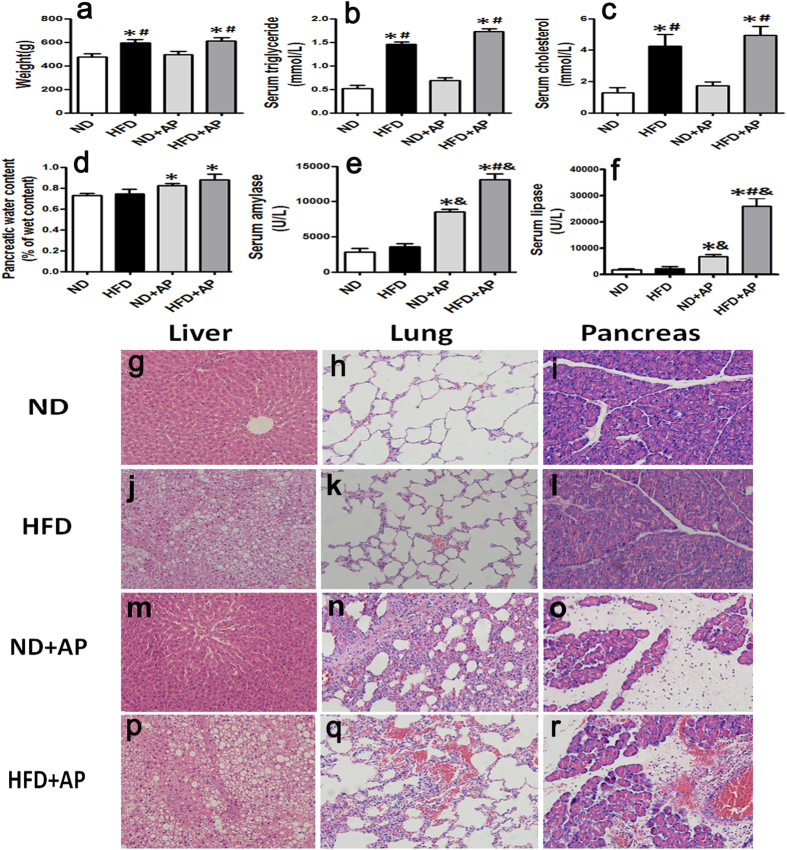
Detection of pancreatitis markers and tissue morphology in the HSAP and NHSAP models. The body weight (**a**), serum triglyceride (**b**), cholesterol (**c**), pancreatic water content (**d**), serum amylase (**e**), and lipase (**f**) were measured after induction of AP. *P < 0.05 vs. the ND group. ^#^P < 0.05 vs. the ND + AP group. ^&^P < 0.05 vs. the HFD group. H&E staining: structure of normal liver (**g,m**), lung (**h,k**) and pancreas (**i,l**); liver structure of rat with HFD (**j**); after establishment of AP, the structure of pancreas and lung in HSAP rats (**r,q**) and NHSAP rats (**o,n**).

A rat model of hepatic steatosis (HS) was established using a high fat diet (HFD) for 60 days. AP was induced surgically as previously described by Aho *et al.*^27^. Compared with the normal diet (ND) group, the body weight (Fig. 1a), serum triglycerides (Fig. 1b), and total cholesterol (Fig. 1c) in the HFD group were increased (P < 0.05). After the induction of pancreatitis, the contents of pancreatic water (Fig. 1d), serum amylase (Fig. 1e), and lipase (Fig. 1f) in the ND +AP and HFD +AP groups were significantly higher than in the ND and HFD groups (P < 0.05). Liver, lung, and pancreatic samples were taken and stained with hematoxylin-eosin (H&E) to assess the establishment of HS and pancreatitis. The structures of normal liver (Fig. 1g,m), lung (Fig. 1h,k), and pancreas (Fig. 1i,l) were clear and complete. In HFD rats, vacuolar lipid droplets were observed in the cytoplasm of the hepatocytes, indicating the establishment of HS (Fig. 1j,p). After establishment of AP, the pancreas of the HFD +AP group exhibited more severe edema, hemorrhage, inflammatory infiltration, and acinar necrosis (Fig. 1r) than that of the ND +AP group (Fig. 1o). Pulmonary hemorrhage, inflammatory cell infiltration, and alveolar epithelial hyperplasia were more severe in the HFD +AP group (Fig. 1q) than in the ND +AP group (Fig. 1n). These histological observations confirmed the successful establishment of a rat model of HSAP and NHSAP.

### AAT is differentially expressed between HSAP and NHSAP rats

**Figure 2:**
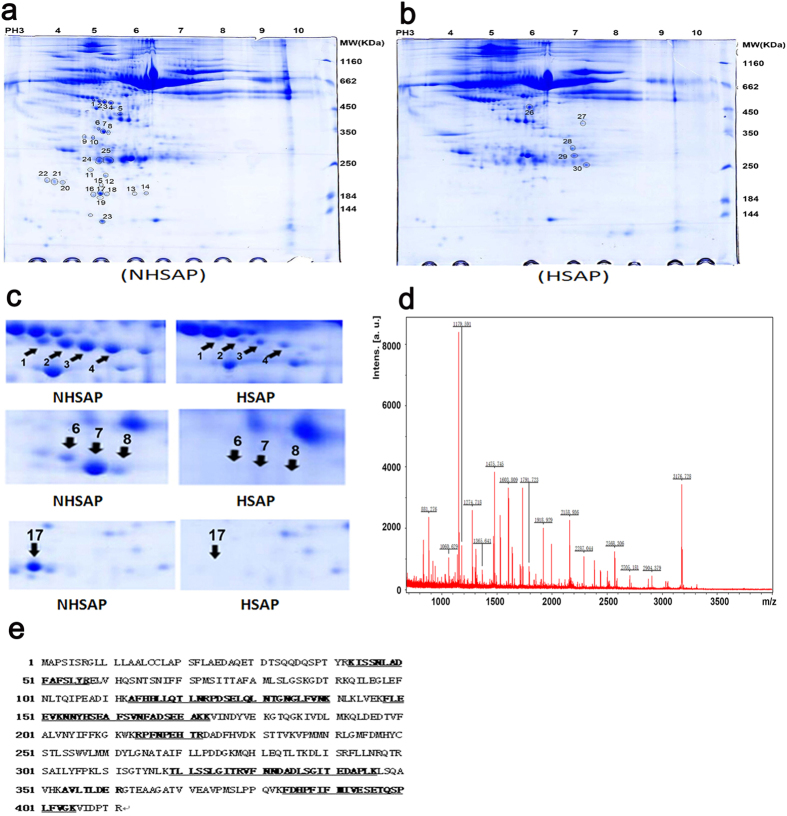
Differentially expressed proteins in the serum of HSAP and NHSAP rat models by proteomics methods. (**a,b**) Representative 2-D maps. (**a**) NHSAP, (**b**) HSAP. (**c**) Eight protein spots were differently expressed between the NHSAP and HSAP groups at 6 h after AP establishment. (**d**) MALDI-TOF-MS identification of protein spot 1, the AAT precursor. (**e**) Protein sequence of the AAT precursor was shown and matched peptides were indicated in bold and underlined.

We used proteomics technologies to assess the spectrum of serum proteins in HSAP (Fig. 2b) and NHSAP (Fig. 2a) rats. Serum proteins were separated first by their isoelectric point (pI) using pH 4 to 10 immobilized pH gradient strips, followed by separation by SDS-PAGE. Molecular weight and pH are indicated at the side and top of gels. The expressions of 30 proteins spots were significantly changed. Ultimately, 18 protein spots were identified by matrix-assisted laser desorption/ionization time-of-flight mass spectrometry (MALDI-TOF -MS), including 4 spots representing up-regulated proteins and 14 spots representing down-regulated proteins (Table 1).

MALDI-TOF-MS analysis identified the protein detected in spots 1 to 4 as the AAT precursor and spots 6, 7, 8, and 17 as apolipoprotein E. These proteins, which had identical molecular weight, but different pI, were found to be significantly lower in the HSAP group than in the NHSAP group at 6 h after surgical induction of pancreatitis (Fig. 2c). We chose to further investigate the expression of AAT, a 52-kD protein previously involved in the pathology of AP^22^,^23^,^24^,^25^,^26^.

### Quantification of AAT, trypsin, and leptin by enzyme-linked immunosorbent assay (ELISA) in rats

**Figure 3:**
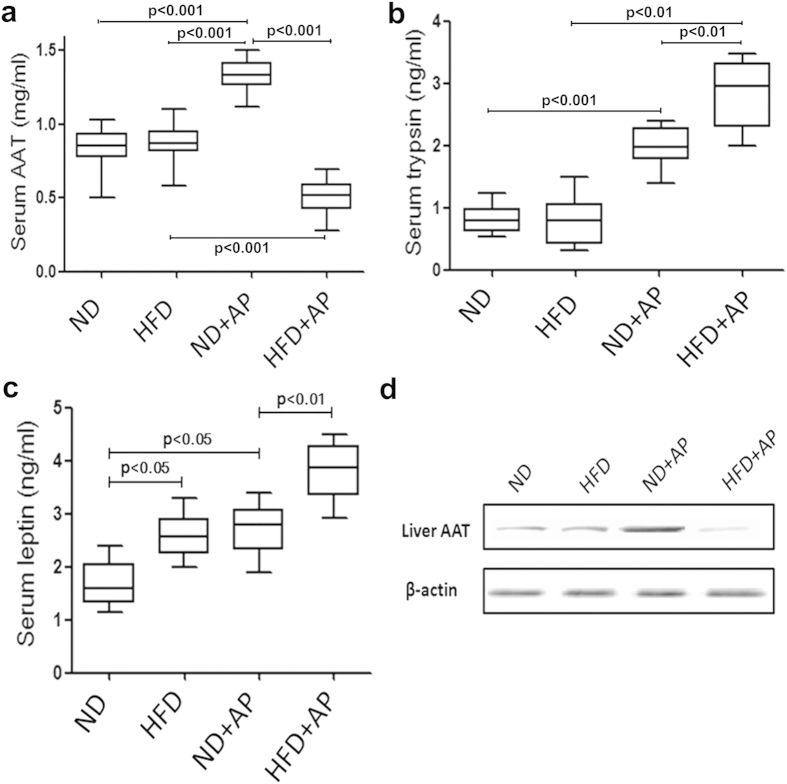
Levels of serum AAT, trypsin, and leptin by ELISA and liver AAT by western blot. The serum levels of AAT (**a**), trypsin (**b**), and leptin (**c**) in rats were measured by ELISA. Liver AAT were quantified by western blot (**d**).

To validate the proteomic identification of AAT as a differentially expressed protein in a rat model of HSAP, we measured the levels of AAT in rat serum by ELISA. The levels of AAT in the serum of HSAP rats were significantly lower than in NHSAP rats (P <  0.001) (Fig. 3a). This was in accordance with the results of mass spectrometry mentioned above. The levels of trypsin and leptin in the serum of HSAP rats were significantly higher than in NHSAP rats (both P < 0.01) (Fig. 3b,c).

### Patient demographics

Between August 2012 and August 2014, 60 patients with HSAP, 60 patients with NHSAP, 60 patients with FLD, and 60 healthy controls were enrolled in the study. The demographic and clinical features of these patients are listed in Table 2.

### Fatty liver is a risk factor for acute pancreatitis

**Figure 4:**
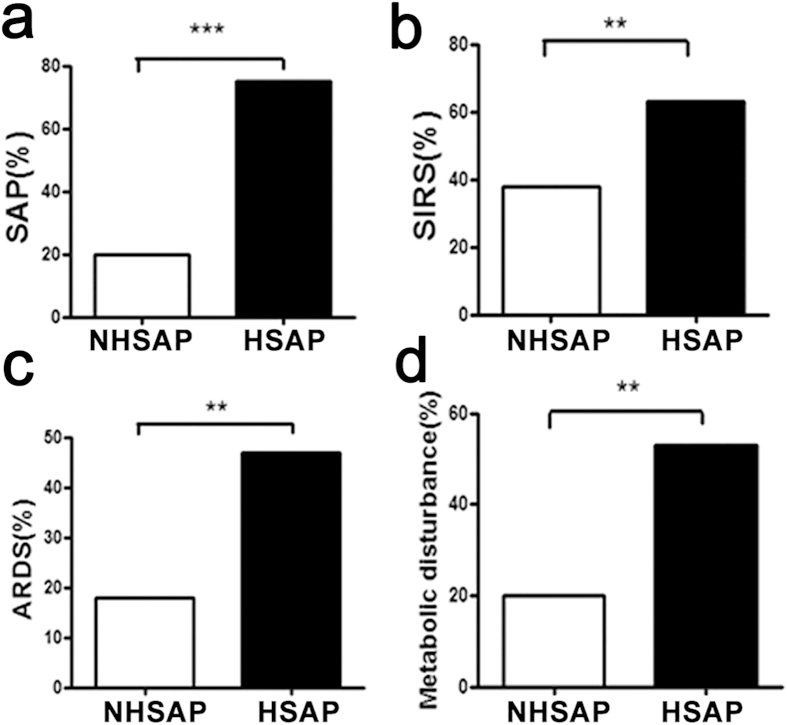
Comorbidities of patients with HSAP and NHSAP. The incidence of SAP (**a**), SIRS (**b**), ARDS (**c**), and metabolic disturbance (**d**) was significantly higher in patients with HSAP (n = 60) compared with patients with NHSAP (n = 60) (chi-square test; **P < 0.01,***P < 0.001).

We found the incidence of comorbidities to be elevated in patients with HSAP. The incidence of SAP, systemic inflammatory response syndrome (SIRS), acute respiratory distress syndrome (ARDS), and metabolic disturbance was significantly higher in patients with HSAP than in patients with NHSAP (P < 0.001, P < 0.01, P < 0.01, and P < 0.01, respectively) (Fig. 4).

### Quantification of AAT by ELISA in human subjects

**Figure 5:**
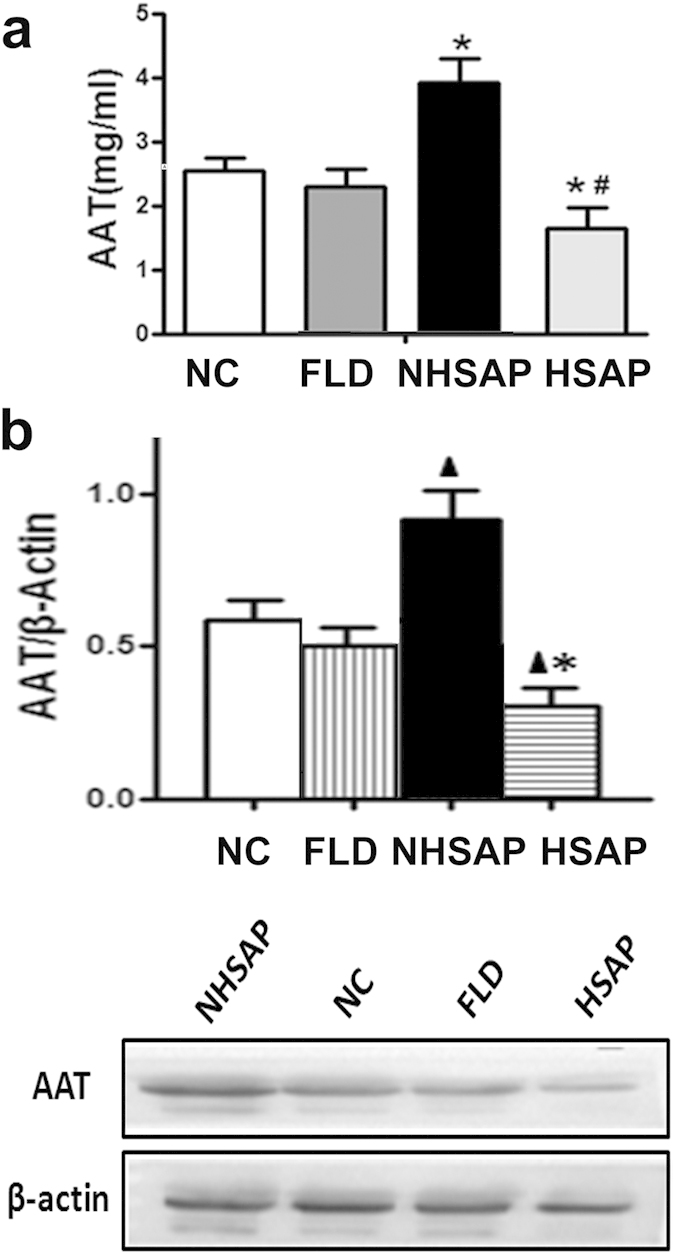
Patient serum AAT levels quantified by ELISA and western blot. (**a**) Serum AAT levels were measured by ELISA in 60 patients with NHSAP, 60 healthy controls, 60 patients with FLD, and 60 patients with HSAP. *P < 0.01 vs. healthy controls. ^#^P < 0.001 vs. the NHSAP group. (**b**) Serum AAT levels were quantified by western blotting in the above four groups. Quantification showing the relative levels of AAT in the four groups. ^▲^P < 0.01 vs. healthy controls. *P < 0.001 vs. the NHSAP group.

To further probe the clinical relevance of the influence of HS on AAT levels in AP, we sought to assess the levels of AAT in the serum of patients with AP and/or HS. AAT levels in the 60 patients with HSAP were 1.63 ± 0.34 mg/ml (range: 0.74–2.59 mg/ml), significantly lower than the AAT levels in the 60 healthy controls, which were 2.54 ± 0.21 mg/ml (range: 2.16–2.90 mg/ml) (P < 0.01). In contrast, AAT levels in the 60 patients with NHSAP were 3.92 ± 0.37 mg/ml (range: 3.1–4.78 mg/ml), significantly higher than in healthy controls (P < 0.01). AAT levels in the 60 patients with HSAP were also significantly lower than in patients with NHSAP (P < 0.001). AAT levels in the 60 patients with FLD were 2.30 ± 0.27 mg/ml (range: 1.81–2.69 mg/ml), and did not differ significantly from the healthy controls (Fig. 5a). These differences were confirmed by western blotting (Fig. 5b).

### AAT levels are correlated with the severity of disease in human HSAP subjects

**Figure 6:**
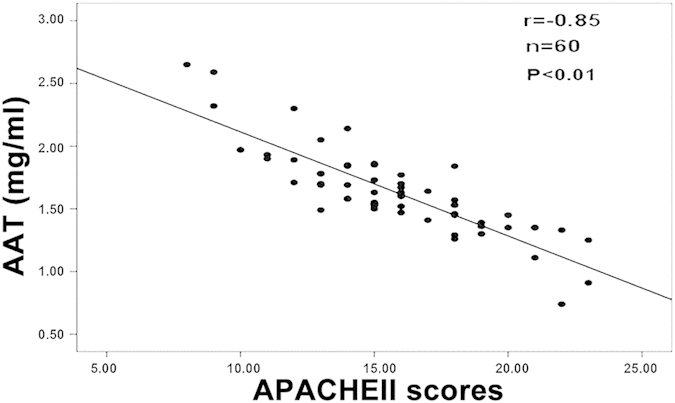
Correlation between serum levels of AAT and disease severity in patients with HSAP. APACHE-II scores were negatively correlated with serum AAT levels (r = −0.85, P < 0.01).

We analyzed the correlation between serum levels of AAT and disease severity in patients with HSAP. We found that APACHE-II scores, measured within 24 hours of hospital admission, were negatively correlated with serum AAT levels (r = −0.85, P < 0.01) (Fig. 6).

## Discussion

HS has been reported to exacerbate AP and to influence the prognosis of pancreatitis. A recent study has demonstrated that HS diagnosed using MRI frequently occurred in a majority of patients with AP. Furthermore, the severity of the HS in MRI was related to the severity of AP^6^. HS is closely related to obesity and obesity or HS is a negative prognostic factor for AP^28^,^29^,^30^,^31^,^32^,^33^,^34^. Consistent with these previous studies, the results of the present study also demonstrated that patients with HSAP experienced a significantly higher incidence of comorbidities than patients with NHSAP.

However, the mechanisms of HS aggravating pancreatitis are not fully elucidated. Proteomics is a new and emerging technology that can identify protein molecules in a high-throughput fashion for serum, biological fluids, and tissues^35^. In the present study, proteomic analysis using 2-D gel electrophoresis was used to identify differentially expressed proteins in the serum of HSAP and NHSAP rat models. We identified 14 down-regulated proteins or isoforms and four up-regulated proteins in HSAP rat models. Serum levels of AAT, an acute-phase reactant previously showed to be elevated in the serum of patients and animals in the early phase of AP^22^,^23^,^24^,^25^,^26^, were found to be significantly depressed in HSAP rats compared with NHSAP rats. A previous study suggested that AAT has significant anti-inflammatory properties by affecting a wide range of inflammatory cells such as neutrophils, monocytes, macrophages, and mast cells^36^. Therefore, decrease of serum AAT levels can lead to the excessive activation of inflammation. In agreement with the present study, the incidence of systemic inflammatory response syndrome (SIRS) in patients with HSAP was significantly higher than in patients with NHSAP.

To further confirm the clinical significance of these findings, we measured AAT levels in the serum of patients with FDL, HSAP, NHSAP, and no pathology. We found serum AAT levels to be significantly lower in patients with HSAP compared with patients with NHSAP. This was consistent with the results of animal experiments. Furthermore, we observed that the depressed serum levels of AAT in patients with HSAP were negatively correlated with APACHE-II scores, potentially highlighting a mechanistic role for AAT in the exacerbation of pancreatitis caused by HS. Actually, AAT deficiency is involved in the pathogenesis of many diseases such as cystic fibrosis^37^, rheumatoid arthritis^38^, diabetes mellitus^39^, viral infection^40^, and other inflammatory diseases. Similarly, AAT deficiency is also involved in the pathogenesis of pancreatitis^41^,^42^,^43^. The present study suggests that decreased serum AAT levels are a consequence of HS and may aggravate the course of pancreatitis.

Serum AAT levels in patients with NHSAP were significantly higher than in healthy controls, suggesting that elevated serum AAT levels may represent a response to pancreatic inflammation. Lempinen also observed that the serum levels of the trypsin2-AAT complex were significantly higher in patients with SAP than patients with mild symptoms^44^. AAT was also elevated in many other diseases including vernal keratoconjunctivitis^20^, hepatic carcinoma, and severe chronic hepatitis^45^, reflecting the protective role of AAT in the tissue response to injury. Circulating AAT levels are reported to increase with greater inflammatory damage, indicating that AAT may represent a useful diagnostic or prognostic marker.

It has been previously reported that serum AAT levels are not changed or only slightly reduced in obese subjects. In the present study, serum AAT levels in patients with FLD were only slightly reduced compared with healthy controls, but this difference was not statistically significant. This observation suggests that hepatic steatosis alone does not cause a significant reduction of circulating AAT levels in the absence of pancreatitis. We hypothesize that pancreatitis impairs the capacity of fatty liver cells to synthesize AAT. In the present study, AAT synthesized and secreted by rats’ liver was detected and the expression of AAT in HSAP rats was obviously lower than in NHSAP rats, which could support the above-mentioned hypothesis. However, it requires further confirmation.

The release and abnormal activation of trypsin has been reported to occur early in the development of pancreatitis^46^. The levels of serum AAT were significantly decreased in HSAP rats, potentially resulting in over-activation of trypsin and imbalance of the trypsin/antitrypsin ratio, and thereby exacerbating AP. Consistent with this view, we found that serum trypsin content in HSAP rats were higher than in NHSAP rats.

AAT is reported to modulate systemic inflammatory responses, reducing production of pro-inflammatory cytokines, blocking leukocyte migration and inhibiting apoptosis^18^,^19^,^20^. However, the precise mechanisms governing regulation of serum AAT levels remain to be determined. Leptin resistance is present in most patients with FLD^47^. Previous studies have indicated that leptin regulates AAT expression via the Jak2-Stat3 pathway in hepatocytes, and the balance between the activities of neutrophil elastase (NE) and its inhibitor AAT in patients with FLD is impaired^48^. Leptin resistance and over-activation of NE may therefore account for the decreased antitryptic activity observed in the serum of HSAP patients. In this study, serum leptin levels in HSAP rats were higher than in NHSAP rats, which suggest that leptin resistance is involved in the pathogenesis of HSAP.

AAT augmentation therapy involves increasing circulating AAT levels to achieve a “protective” threshold^49^. Many studies have explored the clinical application of AAT. AAT levels have been associated with HIV-1infection, diabete smellitus, vasculitis, panniculitis, and hepatitis C infection^50^. In preclinical models of autoimmunity and transplantation, AAT therapy prevents or reverses autoimmune diseases and graft loss^51^, and alleviates ischemia-reperfusion-induced lung injury in human lung transplantation^52^. In addition, purified AAT may be therapeutically effective in acute liver failure^53^, or acute colitis and chronic ileitis^54^. A recently published study suggested that intraperitoneal antiprotease therapy for taurocholate-induced pancreatitis in rats improved survival time^55^. Similarly, administration of human pancreatic secretory trypsin inhibitor prevents the development of experimental acute pancreatitis in rats and dogs^56^. AAT infusion has been established to be a safe therapy; however, to date, no clinical trials of AAT in patients with HSAP have been published. Given that the serum AAT levels in patients with HSAP are depressed, increasing circulating AAT may effectively alleviate the symptoms of pancreatitis in these patients. Our observations, thus, recommend this field of research and potential new strategy in pancreatitis therapy.

## Methods

### Animals

Healthy sex-matched Sprague-Dawley (SD) rats (12 weeks old), weighing 300–350 g, were purchased from the Animal Experimental Center of The Fourth Military Medical University (Xi’an, China) and housed at 25 °C with a 12-h light and darkness cycle. The rats were divided into the following four groups: ND group, ND and surgically induced AP group (ND+AP), HFD group, and HFD and surgically induced AP group (HFD+AP). The ND+AP group was equivalent to NHSAP, while the HFD+AP group was equivalent to HSAP. The ND groups had 5% of their energy from fat, 76% from carbohydrates, and 19% from proteins. The HFD groups received the D12492 feed (Research Diets Inc.) and had 60% of their energy from fat, 20% from carbohydrates, and 20% from proteins. All rats were given free access to water and food. After two months on the HFD, the HS rat model was established. All experimental protocols were approved by the Review Committee for the Use of Animals of the Fourth Military Medical University. All experiments were performed in accordance with NIH guidelines.

### Induction of AP

AP was surgically induced as previously described by Aho *et al.*^27^. Briefly, the rats were anesthetized using a peritoneal injection of 20% urethane. A laparotomy was performed through a midline incision. After exposure of the common bile duct and the pancreas, microaneurysm clips were placed on the bile duct below the liver and around the common biliopancreatic duct at its entry into the duodenum to avoid reflux of enteric contents into the duct. Sodium taurocholate (5%, 0.4 ml/kg, Sigma-Aldrich) was slowly infused into the common biliopancreatic duct. The infusion pressure was maintained below 30 mmHg, measured using a mercury manometer. On completion of the infusion, the two microclips were removed. After ensuring that there was no bile leakage at the puncture site, the abdomen was closed. The entire procedure was performed using sterile techniques.

### HE staining and serum analysis

The rats were sacrificed 6 h after the induction of AP. Venous blood, liver, lung, and pancreas were collected. Tissues were immersed in neutral formalin for 24 h and then dehydrated with ethanol (70%, 80%, 90%, 95%, and 100%) followed by xylene. Tissues were embedded in paraffin and cut into sections, which were used for HE staining (LEICA AUTO STAINER XL). The venous blood was collected in disposable blood collection tubes (separation gel & coagulation accelerator) under sterile conditions and centrifuged at 860 g for 15 min to yield serum. Serum was obtained and used to measure triglycerides, cholesterol, amylase, and lipase using an automatic HITACHI biochemical analyzer.

### Two-dimensional gel and MALDI-TOF-MS identification of differently expressed proteins between rat models of HSAP and NHSAP

Serum samples were collected from HSAP and NHSAP rats 6 h after induction of AP. Highly abundant proteins were isolated using an aurum serum protein mini kit and TCA/acetone precipitation (Bio-Rad), then separated by one-dimensional and two-dimensional gel electrophoresis as previously described^57^. The results of two-dimensional gel electrophoresis were compared using Gel Picker (Bio-Rad). Protein spots were excised and the component proteins were identified by MALDI-TOF-MS using the Triple TOF 5600 (AB Sciex). The experiment was repeated three times. In each experiment, five rats were chosen from each of the HSAP and NHSAP groups.

### Serum AAT, trypsin, and leptin

The rats were sacrificed 6 h after establishment of AP and blood samples were collected from the right atrium through the right jugular, and centrifuged at 860 g for 15 min to obtain serum. Serum levels of AAT, trypsin, and leptin were measured by ELISA (E1697Ra, E1400Ra, E0084Ra, USCN Life Science Inc.) according to the manufacturers’ instruction. All samples were assessed in duplicate. Microplates were read using a Thermo Fisher (MA, USA) microplate reader and AAT levels were calculated from the standard curve by the plate-reader software.

### Western blot of AAT in rat liver tissue

Rat liver tissue samples were lysed in lysis buffer on ice for 20 min. The lysates were clarified by centrifugation at 4 °C for 15 min at 12,000 rpm. After quantification of protein levels, 30 μg of total protein was separated by 10% SDS-PAGE and transferred to nitrocellulose membranes, which were then blocked with 5% fat-free milk at room temperature for 1 h and incubated with anti-AAT antibody (Biorbyt, Cambridge, Cambridgeshire, United Kingdom; #orb10017) at 4 °C overnight. After three washings with TBST, the membranes were incubated with secondary antibody (Zhongshan Golden Bridge Biotechnology, BeiJing) in TBST solution for 1 h at 37 °C, and washed as above. SuperSignal West Pico Chemiluminescent Substrate (Thermo Scientific, USA) was used to visualize the antigens. β-actin (Sigma-Aldrich, USA) was used as an internal control. The experiment was repeated at least three times.

### Human subjects

Between August 2012 and August 2014, patients were enrolled from those admitted to the Department of Gastroenterology at the Fourth Military Medical University XiJing Hospital. Sixty patients with HSAP, 60 patients with NHSAP, 60 patients with FLD, and 60 healthy controls were enrolled in the retrospective study. All groups included 30 male and 30 female patients.

The inclusion criterion for the NHSAP group was based on: 1) acute or persistent abdominal pain ;2) serum amylase and/or lipase activity >3 times the upper limit of normal; and 3) enhanced computed tomography (CT) or abdominal ultrasound suggestive of pancreatitis. Patients with fatty liver, cancer, viral hepatitis, liver cirrhosis, or pregnancy were excluded.

The ratio of liver-to-spleen (L/S) Hounsfield units (HU) <1.0 and liver attenuation <40HU were used for FLD diagnosis and assessment of the severity of liver fat content^58^. L/S ≤0.5 indicated severe fatty liver. Patients with recent heavy alcohol intake were excluded.

The inclusion criteria for the HSAP group were: 1) AP diagnosed within 24 hours of admission; and 2) CT indicated severe fatty liver (L/S ≤ 0.5). Patients with chronic pancreatitis, liver cirrhosis, viral hepatitis, or recent heavy alcohol intake were excluded.

AP was categorized as SAP when :1) associated with local complications (pancreatic necrosis, pancreatic pseudocyst, or pancreatic abscess); 2) complicated with one or more organ failure; 3) APACHE-II score ≥8; and 4) Ranson score ≥3.

The definition of acute respiratory distress syndrome (ARDS) was based on the degree of hypoxemia: mild (200 mmHg < PaO_2_/FIO_2_ ≤ 300 mmHg), moderate (100 mmHg < PaO_2_/FIO_2_ ≤ 200 mmHg), and severe (PaO_2_/FIO_2_ ≤ 100 mmHg); and on four ancillary variables for severe ARDS: radiographic severity, respiratory system compliance (≤40 mL/cmH_2_O), positive end-expiratory pressure (≥10 cm H_2_O), and corrected expired volume per minute (≥ 10L/min)^59^.

The definition of systemic inflammatory response syndrome (SIRS) was based on: 1) body temperature <36 °C or > 38 °C; 2) heart rate > 90 bpm; 3) > 20 breaths per minute; and 4) white blood cell count <4000 cells/mm^3^ or >12,000 cells/mm^3^.

The definition of metabolic disturbances was based on: 1) glucose metabolism disorders (fasting blood glucose >6 mmol/L); 2) lipid metabolism disorders; and 3) electrolyte disturbances.

In accordance with the Helsinki declaration, 60 healthy subjects participated in the experiment voluntarily and signed an informed consent form. They were not suffering from pancreatitis, fatty liver, or other metabolic, acute, or chronic diseases.

All experimental protocols were approved by the Review Committee for the Use of Human Subjects of the Fourth Military Medical University.

### Serum samples

Fasting blood samples were collected at 7 a.m. on the first day after hospital admission. Blood samples (10 ml) were drawn from peripheral veins under aseptic conditions and centrifuged at 860 g for 15 min to yield serum.

### AAT in patient serum samples

Serum levels of AAT were measured by ELISA kits (SEB697Ra, USCN Life Science Inc.) according to the manufacturers’ instruction. All samples were assessed in duplicate. Microplates were read using a Thermo Fisher (MA, USA) microplate reader. AAT levels were calculated from the standard curve calculated by the plate reader software.

### Western blot analysis of AAT in patient serum samples

Serum samples from the 60 patients in each group were pooled and diluted 10 fold in PBS. Serum proteins were separated by 10% SDS-PAGE and transferred to nitrocellulose membranes, which were then blocked with 5% fat-free milk at room temperature for 1 h and incubated with anti-AAT antibody (Biorbyt, Cambridge, Cambridgeshire, United Kingdom; #orb10017) at 4 °C overnight. After three washes in TBST, the membranes were incubated with horseradish peroxidase-conjugated secondary antibody (Zhongshan Golden Bridge Biotechnology, BeiJing) for 1 h at room temperature. SuperSignal West Pico Chemiluminescent Substrate (Thermo Scientific, USA) was used to visualize the antigens. β-actin (Sigma-Aldrich, USA) was used as an internal control^60^. The protein bands were scanned and quantified by densitometric analysis using the Quantity One System (Bio-Rad, Richmond, CA, USA).The experiment was repeated at least three times.

### Statistical analysis

All statistical analyses were performed using SPSS 17.0 (IBM, Armonk, NY, USA). Differences between groups were analyzed using one-way analysis of variance (ANOVA) followed by the Student-Newman-Keuls (SNK) post hoc test. The Pearson correlation analysis was used to assess correlations. Two-sided P-values <0.05 were considered statistically significant.

## Additional Information

**How to cite this article**: Wang, Q. *et al.* Hepatic steatosis depresses alpha-1-antitrypsin levels in human and rat acute pancreatitis. *Sci. Rep.* **5**, 17833; doi: 10.1038/srep17833 (2015).

## Figures and Tables

**Table 1:** Differently expressed proteins in HSAP and NHSAP rat model identified by MALDI-TOF-MS.

NCBI entery	Spot ID	Protein name	Annotation	Protein Score	Protein mass	pI	Coverage
Upregulated proteins							
AAI68872	26	Embryonic ectoderm development	polycomb protein EED	37	50907	6.57	14%
EDM07983	28	rCG53757	intracellular protein transport	33	30828	8.17	14%
EDL81898	29	Glutathione S-transferase,mu type 3, isoform CRA_b	glutathione S-transferase	34	27802	6.13	19%
AAH98746	30	LOC500183 protein	Innate immune related protein	104	26533	7.64	10%
Downregulated proteins							
NP_071964	1	Alpha-1-antiproteinase precursor	acute-phase response, inflammatory response, response to hypoxia and cytokine	191	46278	5.70	33%
NP_071964	2	Alpha-1-antiproteinase precursor	acute-phase response, inflammatory response, response to hypoxia and cytokine	303	46278	5.70	33%
NP_071964	3	Alpha-1-antiproteinase precursor	acute-phase response, inflammatory response, response to hypoxia and cytokine	307	46278	5.70	29%
NP_071964	4	Alpha-1-antiproteinase precursor	acute-phase response, inflammatory response, response to hypoxia and cytokine	305	46278	5.70	38%
NP_001257613	6	Apolipoprotein E precursor	a ligand for the low-density lipoprotein receptor family of proteins	213	35788	5.23	24%
NP_001257613	7	Apolipoprotein E precursor	a ligand for the low-density lipoprotein receptor family of proteins	316	35788	5.23	36%
NP_001257613	8	Apolipoprotein E precursor	a ligand for the low-density lipoprotein receptor family of proteins	203	35788	5.23	28%
NP_543171	11	Fas apoptotic inhibitory molecule 1 (rFAIM)	negative regulation of apoptotic process	38	22690	4.84	79%
CAA69641	12	Hypothetical protein	nucleic acid phosphodiester bond hydrolysis	43	18336	5.98	32%
AAA42018	14	Retinol-binding protein	retinoic acid biosynthetic process	124	20331	5.67	34%
EDM08165	16	Apolipoprotein E, isoform CRA_e	calcium ion binding	41	17827	4.69	12%
EDM08164	17	Apolipoprotein E, isoform CRA_d	Lipid metabolic process	81	17599	4.63	33%
EDL91648	18	Leukotriene B4 12-hydroxydehydrogenase, isoform CRA_b	NADP-dependent enzyme of arachidonic acid metabolism,oxidation-reduction process	34	21344	5.28	20%
EDL96606	23	protein tyrosine phosphatase, receptor type, T (predicted)	protein tyrosine phosphatase activity	34	14418	8.41	8%

Up-regulated proteins exhibited spot intensity at least two-fold increased in HSAP rat serum in comparison to NHSAP rat serum; Down-regulated proteins exhibited at least a two-fold decrease in spot intensity in HSAP rat serum in comparison to NHSAP rat serum.

**Table 2:** Demographic and Clinical characteristics of the study population.

Variable	Nomal (n = 60)	FLD (n = 60)	HSAP (n = 60)	NHSAP (n = 60)	*P* value
Sex(males),n	30	30	30	30	NS
%	50	50	50	50	NS
Age(years)	43 ± 10	45 ± 11	44 ± 13	47 ± 15	NS
Range	(26–62)	(21–64)	(24–67)	(18–74)	NS
BMI(kg/m^2^),mean	21.1	31	29.9	23.6	<0.05^a^
Range	(18–24)	(28–36)	(27–34)	(19–25)	<0.05^a^
AAT(mg/ml)	2.54 ± 0.21	2.30 ± 0.27	1.63 ± 0.34	3.92 ± 0.37	<0.01^b^
Range	(2.16–2.90)	(1.81–2.69)	(0.74–2.59)	(3.1–4.78)	<0.01^b^
Pathogenesis					
Gallstones, n(%)	—	—	22(37)	25(42)	NS
Alcohol, n(%)	—	—	7(12)	9(15)	NS
Greasydiet, n(%)	—	—	26(43)	21(35)	NS
ERCP, n(%)	—	—	2(3)	1(2)	NS
Others, n(%)	—	—	3(5)	4(6)	NS
Complications					
SIRS, n(%)	—	—	38(63)	23(38)	<0.01^c^
ARDS, n(%)	—	—	28(47)	11(18)	<0.01^c^
MD, n(%)	—	—	32(53)	12(20)	<0.01^c^
MODS, n(%)	—	—	12(20)	3(5)	<0.01
Fatty liver CT score (L/S)					
Mean	1.04	0.5	0.4	1.03	<0.05^d^
Range	(1.01–1.06)	(0.3–0.7)	(0.2–0.6)	(1.02–1.05)	<0.05^d^

For^a^ and For^d^: HSAP and FLD group vs. NHSAP and normal group, *P* < 0.05. For^b^ see Fig. 5a and for^c^ see Fig. 4. ERCP, endoscopic retrograde cholangiopancreatography; SIRS, systemic inflammatory response syndrome; MD, metabolic disturbances; MODS, multiple organ dysfunction symdrome; BMI, body mass index; CT, computed tomography; NS, not significant.
